# Ideal Male Umbilicus: An Observational Study of Surface Anatomy and Introduction to the SHAPE Classification

**DOI:** 10.1007/s00266-022-02798-7

**Published:** 2022-02-15

**Authors:** Karissa A. Graham, Ryan J. Livingston

**Affiliations:** 1 Department of Plastic and Reconstructive Surgery, Sunshine Coast University Hospital, 6 Doherty St, Birtinya, QLD 4575 Australia; 2 Department of Medicine, University of Queensland, St. Lucia, QLD Australia; 3 Department of Medicine, Griffith University, South Brisbane, QLD Australia

**Keywords:** Umbilicus, Male, Abdominoplasty, Umbilicoplasty, Aesthetics

## Abstract

**Background:**

The umbilicus is crucial to the aesthetic appearance of the abdomen. With abdominoplasty and umbilicoplasty, placement of the umbilicus is essential and often left at the surgeon’s discretion. This study aims to investigate the ideal male umbilical shape and location by examining photographs of top male models in 2019.

**Methods:**

In this observational study, we examined 81 photographs of top male models to assess different ratios based on anatomical landmarks and umbilical appearance.

**Results:**

The ratio of the distance from the xiphoid to the center of umbilicus (XU) and corresponding distance from center of umbilicus to abdominal crease (UC) had the most reliability (ratio XU/UC, with average measurement: 1.68 ± 0.38), which placed the male navel at a similar position but marginally below the average female umbilicus. Our findings revealed that an oval horizontal is the ideal umbilical shape in males, which differs from what is most aesthetically pleasing in females (oval vertical). In addition, we introduced the SHAPE (Shape, Hood, Adiposity, Protrusion & Position, External piercing) classification for navel appearance to better define the umbilicus and its direct management.

**Conclusions:**

This study establishes that the ideal male umbilicus differs from that of females; it should be placed at the XU/UC ratio of 1.68 ± 0.38 and aim for a horizontal shape with hooding (SHAPE: H II). The SHAPE classification facilitates a logical stepwise approach for the surgeon to refashion the umbilicus.

**Level of Evidence IV:**

This journal requires that authors assign a level of evidence to each article. For a full description of these Evidence-Based Medicine ratings, please refer to the Table of Contents or the online Instructions to Authors www.springer.com/00266.

**Supplementary Information:**

The online version contains supplementary material available at 10.1007/s00266-022-02798-7.

## Introduction

**Fig. 1:**
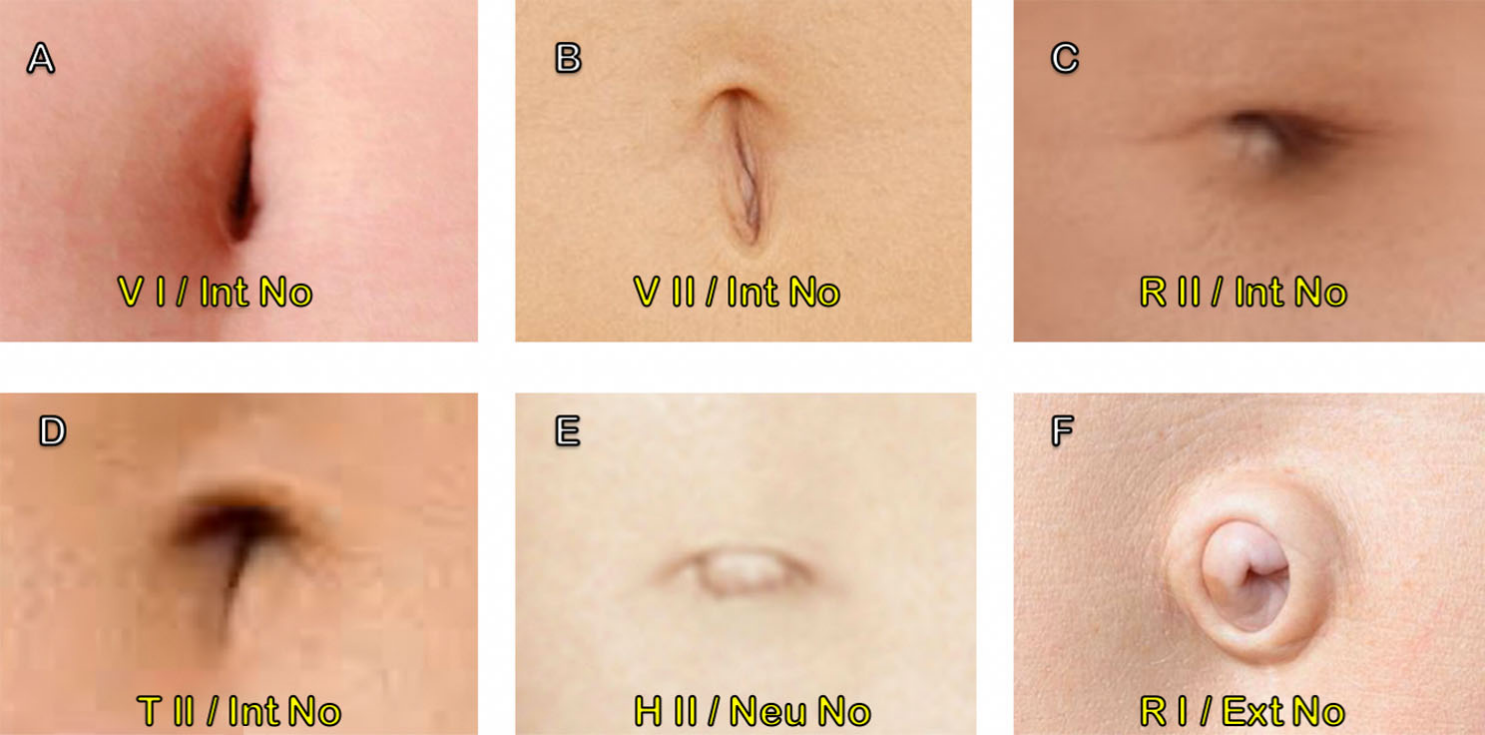
The classification of umbilical shapes based on the Craig classification system [4]. **A** “Vertical lozenge” with no superior hooding; **B** “oval vertical” with superior hooding; **C** “round” with superior hooding; **D** “T-shaped” with superior hooding; **E** “oval horizontal” with superior hooding and **F** “protruded/outie” with superior hooding. Yellow text is the umbilicus based on the SHAPE classification. Of note “/” has been used in place of adiposity, as a proper assessment of this factor is not possible from these photographs

The male umbilicus is an essential landmark crucial to the overall aesthetic appearance of the abdomen [1]. Lately, umbilicoplasty (unrelated to abdominoplasty) has gained widespread recognition, as distortion and dysmorphia of the navel are perceived as unattractive [2]. The concept of an ideal umbilicus is influenced by society, and contributing factors include age, ethnicity, and personal preference [3]. Craig et al. [4]. outlined a classification system (Fig. 1) used for the female umbilicus and that the ideal position is located at the xiphoid–center of umbilicus/center of umbilicus–abdominal crease (XU/UC) golden ratio or divine proportion (1.618).

This paper introduces a more comprehensive classification system, which was clinically used by the senior author to guide operative decision-making. The SHAPE classification, conceptualized by the last author, is straightforward and extremely useful in considering patients’ wishes when outlining a surgical management plan for umbilicoplasty. The SHAPE classification system identifies Shape, Hood, Adiposity, Protrusion, Position, Ptosis, and External piercing (Table 1) as essential components needed to define and direct the reconstructive planning of an umbilicoplasty for both males and females. Besides, the attempt to change the umbilicus to a more pleasing appearance could be negotiated through this simple framework by discussing with patients (Table 2).

**Table 1:** The SHAPE classification of an umbilicus. It is recommended to assess individual components of the classification with a patient standing, which allows gravity to exert influence on the region

	Description			
Shape	V (vertical oval)	H (horizontal oval)	R (round)	T (T-shape)
Hood	Type I (no hood)	Type II (superior hooding covering <10%)	Type III (superior hooding covering 10–30%)	Type IV (superior hooding covering >30%)
Adiposity	Type a (tendinous insertions of the rectus visible)	Type b (linea alba visible)	Type c (round abdomen)	Type d (ptotic abdomen)
Protrusion	Int (internal)	Neu (neutral)	Ext (external)	
Position	Midline	Right-sided	Left-sided	
Ptosis	Type 1 (normal position at the golden ratio or no descent of umbilicus)	Type 2 (ptosis but the umbilicus lies above the abdominal crease)	Type 3 (ptosis but the umbilicus lies below the abdominal crease)	Type 4 (Type 3 + umbilicus angled to the floor)
External piercing	N (no)	Y (yes)

**Table 2:** The SHAPE classification practical usage

Shape	The shape can be adjusted to mirror the population’s desired “normal” for aesthetic appearance or tailored to a patient’s preference; this could include a patient’s choice to forgo or remove the umbilicus. SIZE—the size can also be considered under shape; it can be decreased or increased to fit the optimal size of an umbilicus required. Besides, the vertical height and horizontal width should be considered, along with the shape. Both the abdominal skin and umbilical skin can be adjusted to change shape and size. The two desired shapes for the neo-umbilicus can be designed with differing incisions. For horizontal oval shape, the incision is a horizontal crescent moon (concavity facing superiorly) or an “H” shape depending on requirements. The superiorly based flap is usually larger than the inferiorly based flap, if it is present. The dimensions are based on the native umbilical size. A superior flap is excised from the oval to achieve the desired hooding (see Videos 1 and 2). For a V shape, a “Y” incision is made, which allows for a superior flap for hooding.
Hood	A ptotic hood can be revised and a pexy suture placed in the superior umbilicus and down to the abdominal wall. This will alter the position of the hooding to a more natural appearance and accentuate concavity above the hood. A superior abdominal flap can be fashioned to increase hooding (see Video 2).
Adiposity	Adiposity around the umbilicus can be manipulated with a defatting procedure to simulate the linea alba above the hood or by thinning the abdominal flap and removing adiposity around the umbilicus. As many first-world countries are moving toward obesity, perhaps these techniques could be used as an adjunct to liposuction, which may already be planned for utilization in the periumbilical area. This procedure should be performed within the constraints of what perfusion to the skin flap allows. Similarly, fat grafting could be used to accentuate a feature or cover an undesirable attribute underlying thin skin.
Protrusion, Position, and Ptosis	Pexying sutures to decrease protrusion are usually performed by using a suture from the native umbilicus down to the abdominal wall. The umbilical position is set at the golden ratio, within the limits of what the umbilical stalk length and vascularity of the umbilicus allow, with the male umbilicus lying slightly more inferior if aiming at a “normal position” (see Table 3). Remember to adjust for any scoliosis, malalignment, and difference in limb length (this should be planned while the patient is standing before surgery), by premarking from the sternal notch through the xiphisternum to the mid-point of the pubis while the patient is standing in a sagittal orientation; this will guide the centrality of the umbilical placement. Ptosis can be overcome by repositioning of the new umbilicus through the abdominal flap and using pexy sutures to secure the abdominal flap superior to the umbilicus to the abdominal wall (see Video 3). It is crucial not to put undue tension on the stalk to maintain vascular supply, even if it means the position is not quite ideal.
External piercing	Piercings can be removed at the patient’s discretion. If patients want to retain their piercing, it must be considered in the umbilicoplasty design to avoid mispositioning. Perhaps, it might be easier to remove and ask patients to get the navel re-pierced in the future.

Evaluation of the male umbilicus is crucial because of the anatomical differences between males and females. International Society of Aesthetic Plastics Surgeons (ISAPS) and National Plastic Surgery Society members reportedly performed 888,712 abdominoplasties in 2018, with 76,325 (8.6%) on males [5]. It is the seventh most common surgical cosmetic procedure performed on males [5]. The majority of papers written on the ideal umbilicus are based on analysis of a female [3, 4, 6, 7] with a few papers comparing male to female umbilicus [2, 8, 9] and none solely focusing on the male umbilicus. Freeman and Wiemer [9] advise no sexual differences between male and female umbilical appearance, while others claim differences in shape and position [2, 8].

The ideal shape and positioning differences described in the literature demonstrate the necessity for further research to be done. This study aims to assess the ideal male umbilical shape and location by examining photographs of top male models in 2019 to elucidate the differences between male and female. We hypothesized that the positioning of a male umbilicus would be different from that of a female given the differences in skeletal structure and propensity to store the abdominal wall fat, and thus, the aesthetically pleasing shape would also differ.

## Methods

### Study Design

To assure continuity of results and accurate comparison between male and female ideal umbilicus, we used a methodology like Visconti et al. [3], who examined the ideal female umbilicus. In this observational study, we analyzed 81 online photographs of top male underwear models that were published from January 1 to December 31, 2019, which were obtained from top online male magazines and underwear merchants such as Calvin Klein, Emporio Armani, Hugo Boss, Dolce & Gabbana, GQ.com, and mensvariety.com. The study was based out of Queensland, Australia, but the male models originated from all continents, except for Antarctica, thereby ensuring a broad range of races/ethnicity. We only selected high-definition, color photographs with good light exposure, anterior view, and the model was standing in a neutral orthostatic position with the entire abdomen exposed (from nipples to umbilical crease and across to both flanks). As we could not standardize the photographs, we excluded photographs with any obstruction to viewing the abdomen (i.e., clothing, tattoos), black and white, raised arms, unnatural pose, or blurred. The average age of the models was 31 (range: 19–48) years and average height 185.2 (range: 167–196) cm.

### Photograph Analyses

**Fig. 2:**
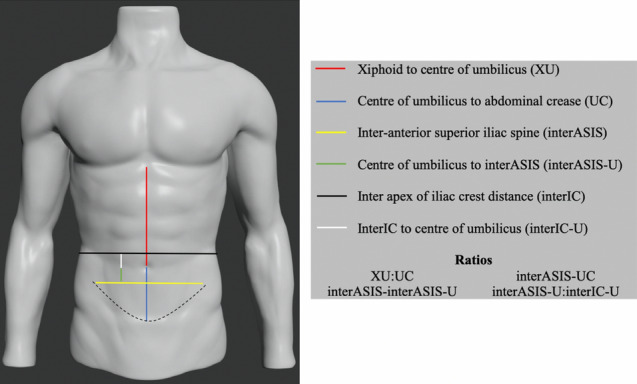
A sketch outlining the male anterior abdominal framework and the ratios obtained from each photograph. Red line, xiphoid to the center of umbilicus (XU); blue line, the center of the umbilicus to the abdominal crease (UC); yellow line, the inter-anterior superior iliac spine line (interASIS); green line, the center of the umbilicus to the interASIS line (interASIS-U); black line, the inter apex of iliac crest line (interIC); and white line, the interIC to the center of the umbilicus (interIC-U) Source of the male torso drawing: RenderHub. Male Torso Mannequin 3D Model. March 5, 2020 10:32 EST. Available at: https://www.renderhub.com/dcbittorf/male-torso-mannequin. Accessed June 12, 2020

We performed two analyses of the navel position in top male underwear models in 2019. In the first analysis, we determined the following four proportions from the photographs: (i) xiphoid–center of umbilicus/center of umbilicus–abdominal crease (XU/UC); (ii) inter-anterior superior iliac spine line/umbilicus–abdominal crease (interASIS/UC); (iii) interASIS/umbilicus–interASIS (interASIS/interASIS-U); and (iv) umbilicus–interASIS/inter iliac crest line–umbilicus (interASIS-U/interIC-U), as shown in Fig. 2. The abdominal crease is defined as the line where the abdomen transitions into the mons. Measurements were performed using the Ruler tool in Adobe Photoshop^®^ ver. 20.0.5 by Adobe Inc. in San Jose, CA, USA.

In the second analysis, we used Zoom tool in Adobe Photoshop^®^ to magnify the photograph and then use the SHAPE classification system to identify the navel shape. Besides, we measured the vertical/horizontal (V/H) ratio for each umbilicus with the Ruler tool in Adobe Photoshop^®^.

### Survey to Analyze the Male Umbilical Shape

We conducted an online questionnaire-based survey to analyze the male umbilical shape. The survey was emailed to 150 people on the primary author’s contact list. The survey closed 4 weeks after it was distributed. The total response rate was 74% (111 respondents of 150 surveys distributed). There were 108 valid responses [68 female, 40 male; average age: 39 (range: 24–79) years] with 50 people from Australia, 52 from Canada, 4 from the USA, and 2 from the Philippines. Three participants did not complete the survey and were, therefore, excluded.

The questionnaire was based on the survey distributed by Visconti et al. [3] for female umbilical preferences. It asked for the participant’s age, sex, and country of residence, followed by two questions: (i) Of the photographs below, which one has the MOST harmonious, natural-looking and aesthetically pleasant navel?; and (ii) Of the photographs below, which one has the LEAST harmonious, natural-looking and aesthetically pleasant navel?. The questions were accompanied by 6 photographs, selected from a pool of 81 photographs used in this study, showing different umbilical shapes in males. All the photographs depicted the anterior abdominal wall from the nipples to the pubic symphysis.

The survey itself was not about validating the classification but outlining an ideal male umbilicus. The classification identifies a useful stepwise approach to consider the umbilicus and how the authors put that into practice.

### Statistical Analysis

The ratios were recorded as numerical values, and the mean values with standard deviations were calculated. Categorical data were collected to analyze the SHAPE classification, and a percentage was used to determine the frequency of umbilical shapes. Survey responses were recorded from the two questions as categorical data, and a percentage was calculated. Statistical analyses were performed using Microsoft^®^ Excel (ver. 16.16.22; Microsoft, Albuquerque, NM).

## Results

### Study Cohort

We examined a total of 81 photographs of male models.

### Photograph Analyses

**Fig. 3:**
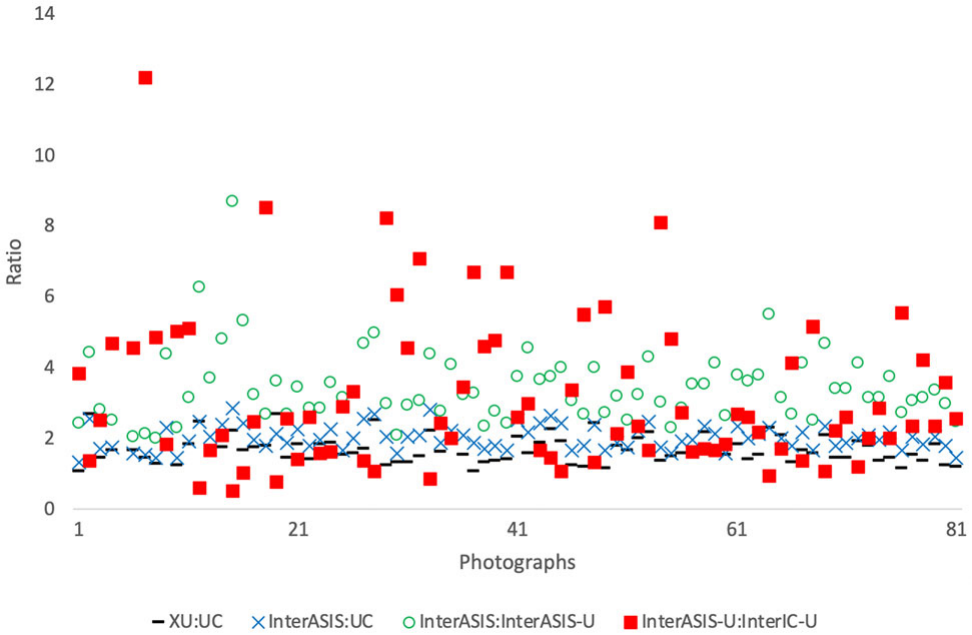
The scatter plot of the umbilical ratios measured in all photographs analyzed. XU, xiphoid to the center of umbilicus; UC, the center of the umbilicus to the abdominal crease; interASIS-U, the center of the umbilicus to the interASIS line; interIC-U, the interIC to the center of the umbilicus

Analysis of the ratios based on anatomical landmarks showed the mean xiphoid–center of umbilicus: center of umbilicus–abdominal crease (XU/UC) ratio was 1.68 ± 0.38, mean inter-anterior superior iliac spine line/center of umbilicus–abdominal crease (interASIS/UC) ratio was 2.02 ± 0.35, mean interASIS/umbilicus–interASIS (interASIS/interASIS-U) ratio was 3.43 ± 1.04, and mean center of umbilicus–interASIS/inter iliac crest line–center of umbilicus (interASIS-U/interIC-U) ratio was 3.41 ± 3.03. Table 3 and Fig. 3 provide the summarized results of ratios. The range and standard deviation for interASIS/interASIS-U and interASIS-U/interIC-U ratios were high, suggesting that these ratios may not be as reliable.

**Table 3:** Summary of Ratios of the Male Umbilical Position

Ratio	Mean ± SD	Range
XU:UC	1.68 ± 0.38	1.06–2.70
interASIS:UC	2.02 ± 0.35	1.26–2.89
interASIS:interASIS-U	3.43 ± 1.04	1.90–8.71
interASIS-U:interIC-U	3.41 ± 3.03	0.54–22.42

Mean ± standard deviation and range of values from minimum to maximum. XU, xiphoid to the center of umbilicus; UC, the center of the umbilicus to the abdominal crease; interASIS-U, the center of the umbilicus to the interASIS line; interIC-U, the interIC to the center of the umbilicus.

The analysis based on the SHAPE classification revealed that of the 81 navels examined, 2 navels (2.5%, *n* = 81) were vertical oval (SHAPE: V I a Int N), 5 (6.2%, *n* = 81) were vertical oval with hooding (SHAPE: V II a Int N), 34 (42.0%, *n* = 81) were round with hooding (SHAPE: R II a Int N), 3 (3.7%, *n* = 81) were T-shaped with hooding (SHAPE: T II a Int N), 29 (35.8%, *n* = 81) were horizontal oval with hooding (SHAPE: H II a Neu N), and 8 (9.8%, *n* = 81) were protruding (SHAPE: R II a Ext N).

**Fig. 4:**
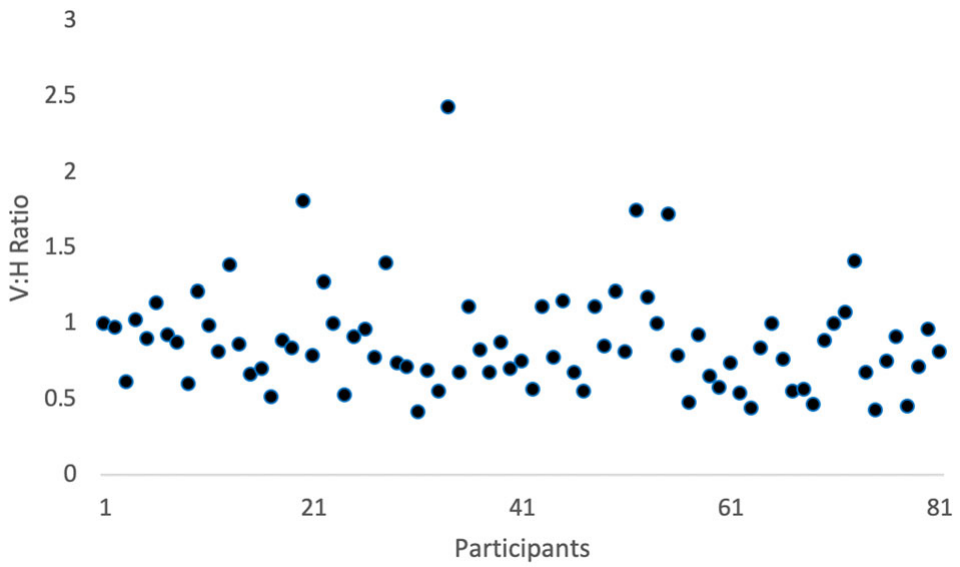
The vertical to horizontal umbilicus (V/H) ratio for all photographs

Figure 4 shows the V/H ratio for each participant. In this study, the mean V/H ratio was 0.88 ± 0.34 (range: 0.41–2.42), suggesting that an umbilicus marginally longer in the horizontal direction was more common.

### Survey to Analyze the Male Umbilical Shape

Of 108 respondents, the most commonly selected shape for the most aesthetically pleasing umbilicus was the horizontal oval (SHAPE: H II a Neu N) with 58 votes (54%, *n* = 108), followed by round with hooding (SHAPE: R II a Int N) with 20 votes (19%, *n* = 108). There was an equal number of respondents who selected vertical oval with (SHAPE: V II a Int N) and without hooding (SHAPE: V I a Int N) for the most aesthetically pleasing; 8 votes each (7%, *n* = 108). Twelve participants (11%, *n* = 108) voted for the T-shape (SHAPE: T II a Int N). Only 2 respondents rated the protruded umbilicus (SHAPE: R II a Ext N) as the most aesthetically pleasing (2%, *n* = 108). Interestingly, when looking at just the male survey respondents (the less dominant gender of the respondents), the most aesthetically pleasing umbilicus was still the horizontal oval (35%, *n* = 40), followed by round with hooding (25%, *n* = 10).

The shape that was most commonly selected, by the 108 respondents, as the least aesthetically pleasing umbilicus was the protruding umbilicus with 52 votes (48%, *n* = 108), followed by vertical oval without hooding (SHAPE: V I a Int N) with 28 votes (26%, *n* = 108), and vertical oval with hooding (SHAPE: V II a Int N) with 16 votes (15%, *n* = 108). Round with hooding (SHAPE: R II a Int N), T-shape with hooding (SHAPE: T II a Int N), and horizontal oval (SHAPE: H II a Neu N) were least commonly chosen as the least aesthetically pleasing; 6 votes (5%, *n* = 108), 4 votes (4%, *n* = 108), and 2 votes (2%, *n* = 108), respectively.

While the most aesthetically pleasing navel was the horizontal oval [SHAPE: H II a Neu N (54%)], the least was a protruding umbilicus [SHAPE: R II a Ext N (48%)].

## Discussion

The umbilicus is an aesthetic unit of the abdomen, with many factors contributing to its appearance, including Shape, Hooding, Adiposity, Protrusion, Position, and External piercing. There is a lack of consensus on what is considered ideal and is often left to the surgeon’s sense of beauty [3].

In our study, the male umbilical position [xiphoid–center of umbilicus/center of umbilicus–abdominal crease (XU/UC) ratio 1.68 ± 0.38] was similar to previous studies on ideal female umbilical position [xiphoid–center of umbilicus/center of umbilicus–abdominal crease (XU/UC) ratio 1.62 ± 0.16] [3]. Other studies have looked at female umbilical placement [10–13], but no other study has looked specifically at ideal male umbilical placement. Yu et al. [8] found that the male umbilicus anatomically was located ~1cm inferior to females. Our study further supports this by adding a marginally inferior placement is more aesthetically pleasing.

Our results suggest the xiphoid–center of umbilicus/center of umbilicus–abdominal crease (XU/UC) ratio may be the most reliable ratio for male umbilical placement given the small standard deviation size (1.68 ± 0.38). Further studies make use of the vertical ratios to estimate umbilical placement as well as looking at ethnicity. The following studies examined the ratio of xiphoid process to umbilicus and umbilicus to pubic symphysis: a study of Indian females found the ratio was 1.6 [10], a study of 100 Korean women showed a ratio of 1.07 [11], and a further study of 100 Latin-American nulliparous women found a ratio of 1.10 [12]. This Latin-American study also suggested that the umbilicus lies lateral to the midline axis in 80% of cases [12]. Although these were not the landmarks used in this study, it suggests that the vertical measurements may be the most reliable. It also elucidates an important point regarding race and its impact on umbilical placement. This was not an outcome measure that was specifically analyzed in this study. The literature does suggest that race does affect umbilical placement with one study suggesting that African–Americans have a lower lying umbilicus compared to Caucasians [13].

Of note, measurements for the described ratios are realized on a two-dimensional photograph. Transferring this knowledge to the clinical setting warrants remembering two crucial points. First, the most reliable landmark inferiorly is the umbilical crease. As this is not a bony landmark, it can be changed with soft tissue position; thus, this necessitates it to be marked preoperatively with patients in the standing position. Second, the measurements should be obtained in a straight line parallel to the abdomen and not conforming to the three-dimensional anatomy of the abdomen, which could artificially change the ratio designed for use in a two-dimensional setting.

Interesting bony landmarks lack reliability when assessing the umbilical position [3]. When one considers the pelvis, however, it does not surprise that a difference exists in the umbilical position found in this study when considering the pelvis differences between sexes, given the physiological adaptions for childbirth [14].

### Shape and Hood

Our study confirmed a difference between male and female umbilical shape. The most common (42%) male umbilical shape was round (SHAPE: R II a Int N), while the least common (2.5%) was vertical oval with hooding (SHAPE: V II a Int N). This is different to the leading female umbilical shape, the “oval vertical” with superior hooding (SHAPE: V II / / /), while a “protruding” navel (SHAPE: / / / Ext /) was the least common [3].

This study demonstrated that the most aesthetically pleasing navel in males was horizontal with hooding. Lee et al. [7] concluded that the ideal female umbilicus was an oval vertical with or without hooding, Visconti et al. [3] found the oval vertical with hooding, and Craig et al. [4] found either T-shaped or vertical.

**Fig. 5:**
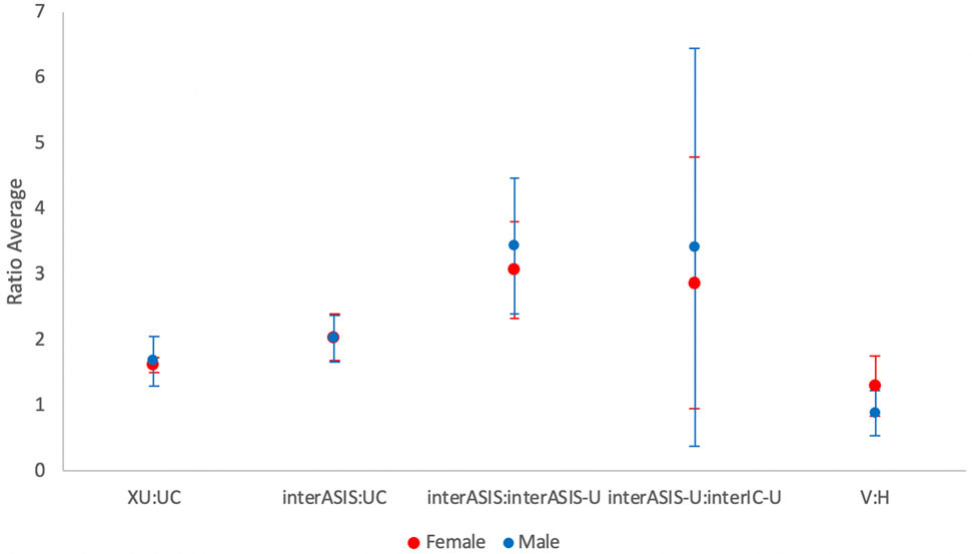
Comparison of the mean ratios ± standard deviation for the male umbilicus from this study and the female umbilicus from Visconti et al. [3]

Regarding the V/H ratio of the umbilicus, our study reported that an umbilicus marginally longer horizontally than vertically is more common, corroborating the horizontal shape (SHAPE: H) being the more aesthetically pleasing navel as described earlier but in contrast to that reported for a female umbilicus that tends to be more vertical than horizontal [3]. Figure 5 shows the comparison of the ratios for males performed in this study and that for females in Visconti et al. [3].

### Adiposity

As our study cohort comprised professional models, adiposity was not representative of a true cross section of a standard population.

Adiposity adds an extra dimension to the abdomen, which is common in a population with increasing BMI. Like nipple ptosis in the breast, the umbilical stalk ptose with the pannus over time; although it is not addressed in this study, it is evident for anyone undertaking abdominoplasty in an aging or weight loss population. The ideal umbilicus in this study addresses the ideal aesthetic position of an umbilicus that we should strive for and can clinically be used much in the same way nipple position is returned to an ideal position in a mastopexy.

### Protrusion, Position, and Ptosis

Only 8 (9.8%, *n* = 81) of the photographs analyzed demonstrated a protruding umbilicus (SHAPE: R II a Ext N), suggesting that it is not found very commonly. Furthermore, our study confirmed the least pleasing umbilicus in males was one that was protruding. The protruding umbilicus is also most commonly reported as unappealing in females [3, 4]. The umbilical position should be closely related to the golden ratio, as highlighted by our results. Although our study population had no ptosis of the umbilicus, it should be considered in this category.

### External Piercing

None of the models in this study had an external piercing, which suggests it is not considered attractive or commonplace in male models. The piercing, however, can be removed during umbilicoplasty if desired. In our experience, piercings alter the complexity of surgical decision making. In practice, the piercing is usually removed and can be easily re-pierced after the surgery at the patients’ discretion. It is important to discuss a piercing in the outpatient setting, while forming the surgical plan.

See Table 2 and Videos 1–4 for practical applications and surgical techniques of the SHAPE Classification. By analyzing the umbilicus using the SHAPE classification, a surgeon breaks the anatomical landmark into distinct defining characteristics and, therefore, allows for focused alterations that result in a more pleasing and standardized outcome.

**Fig. 6:**
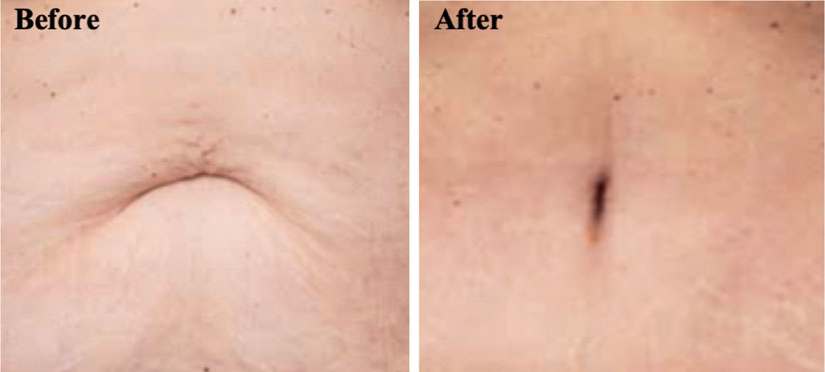
Use of the SHAPE classification in a female to reconstruct the umbilicus in an abdominoplasty procedure. The “Before” and “3 months After” photographs demonstrate conversion from SHAPE: H IV d I N (horizontal oval with >30% hooding) to SHAPE: V I c I N (vertical oval with no hooding). During the preoperative discussion, the surgeon provided the patient with photographs of common umbilical shapes and asked her to select her “ideal.” Following this, a discussion regarding her current umbilical SHAPE and what she desired postoperatively ensued. She desired a vertically oriented umbilicus, with no hooding, minimal resection of the surrounding adiposity, and maintain internal protrusion. There was no piercing to consider. Thus, the senior surgeon placed the umbilicus near the golden ratio (with care to avoid excess tension on the umbilical stalk), in a “Y”-shaped skin incision (allowing a superior flap and suture line to be below the plane of the abdomen, hence, concealing the scar). Minimal fat resection of 3–4 cm of the superior abdominal flap, a loose superior and a tight inferior pexy suture to the abdominal wall, and skin closure completed the procedure. The umbilicus was splinted loosely with a Vaseline-impregnated gauze for 2 weeks

**Fig. 7:**
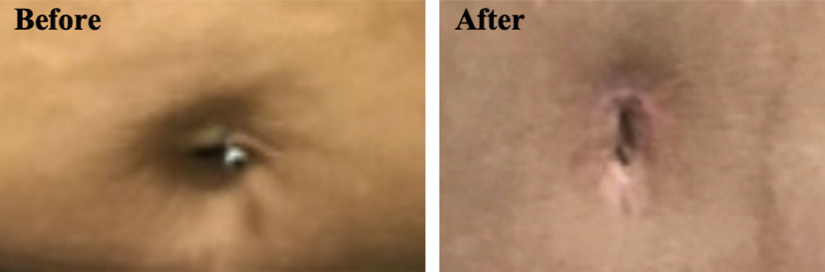
Demonstration of the SHAPE classification in a patient with a piercing. She presented with an implant-based reconstruction, but was not satisfied with the result and so a left DIEP breast reconstruction was performed. A preoperative discussion revealed that she wished for a vertical oval umbilicus with no hooding and removal of the piercing. The “Before” photograph demonstrates SHAPE: H III c I Y (horizontal oval with some hooding and a piercing). The “After” photograph, taken at 6 weeks demonstrate SHAPE: V I b I N (vertical oval with no hooding and removal of piercing)

**Fig. 8:**
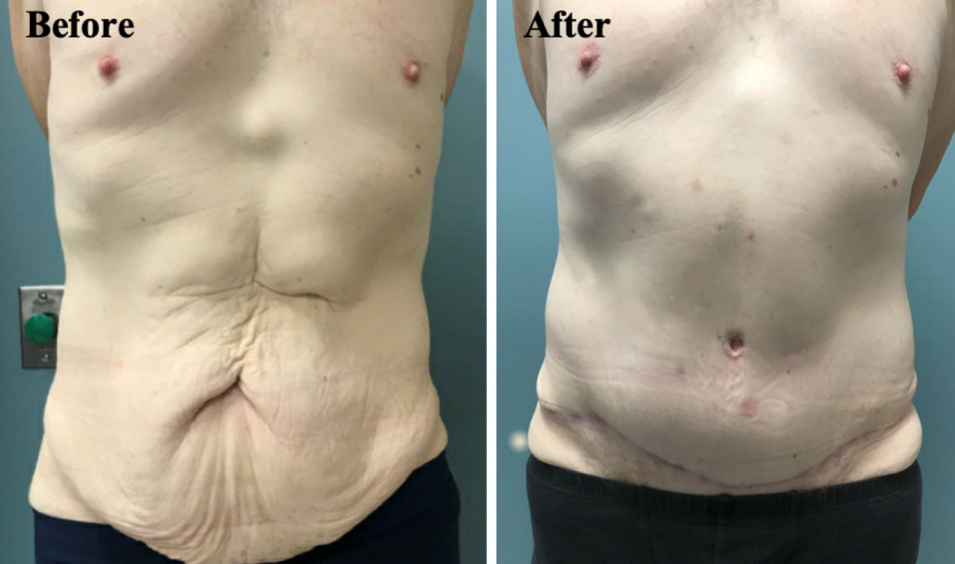
Male abdominoplasty using the SHAPE classification to reconstruct the umbilicus. His “Before” photograph demonstrates SHAPE: H IV d I N (horizontal oval with significant hooding in a ptotic abdomen). Intraoperatively, a horizontal crescent moon (concavity pointing superiorly) shape allowed a superior flap to be sutured in to the umbilicus and provide some subtle hooding. This was further accentuated by a linea alba cutaneous pexying suture. In addition, some sculpting of the fat of the superior abdominal flap provided a more defined linea alba appearance. The “After” photograph 8 weeks postoperatively demonstrates the umbilicus changing to SHAPE: H I b I N (horizontal oval with mild hooding). See Videos 1–4 for a demonstration of the intraoperative steps using the SHAPE classification as a framework for a male abdominoplasty patient

One of the limitations of this study was that it did not correlate height, weight, and age with the position and shape of the umbilicus. Whether height is a relevant factor remains debatable [2, 15]. Age may play a role in influencing the shape of an umbilicus, with a transition from a more vertically orientation to a T or horizontal shape with aging, but this is likely a result of increasing BMI as people age [2]. Evaluation of adiposity and piercings was limited in this study given our population cohort. Furthermore, as models were selected from top male magazines written in English, the findings could be biased toward certain races/ethnicity with different ideals of attractiveness. We feel, however, that this is not a study limitation, but more a limitation on society’s views on male models and the discordance that might exist between models and a more generic population. Accordingly, we have added a couple of case examples to demonstrate how the last author would discuss adiposity with the patient for the readers’ information (Figs. 6, 7, 8). It is impossible to determine one globally acceptable ideal.

As the photographs used in this study were published in magazines and on websites, they were likely photoshopped. We accepted this likelihood as we aimed to determine the ideal position and shape as determined by society, which we presumed the photoshoppers were also striving for. Plastic surgeons also strive to alter and reform not only for function, but aesthetic beauty, and like the photoshopper, aim for the most pleasing form.

The findings of this study are instrumental in considering the most “ideal” shape for a male patient and can guide discussions regarding patients’ wishes and surgical constraints. Body habitus and local/regional features may not allow every surgical candidate to attain an ideal result; however, it allows a framework to work toward an aesthetic goal. Classifications assist operative decision-making, but all treatments are individualized to patients and the complexity of their body, such is the artistry of Plastic and Reconstructive Surgery.

## Conclusions

There are definite differences between male and female umbilicus that have been outlined in this study. We have shown that the xiphoid–center of umbilicus/center of umbilicus–abdominal crease (XU/UC) ratio is the most reliable to ascertain the best umbilical position in males, with an average of 1.68. A surgeon should consider using the horizontal oval (with the horizontal dimension 110–120% larger than the vertical dimension, V/H ratio 0.88, which is interestingly opposite to female) with superior hooding (SHAPE: H II) to create the most pleasing umbilicus in males and consider using the SHAPE classification to define and plan surgical umbilicoplasty. Nevertheless, this should not replace a discussion between the surgeon and the patient regarding the patient’s preferences. As this study has shown, the pleasing umbilicus to the population at large may differ from what a patient’s individual goals are for their umbilicoplasty.

## Supplementary Information

Below is the link to the electronic supplementary material.

Supplementary file1 (MOV 216387 KB)

Supplementary file2 (MOV 11732 KB)

Supplementary file3 (MOV 17361 KB)

Supplementary file4 (MOV 25597 KB)
